# Evaluation of crizotinib as radiosensitizer in sacral chordoma cells: effects of combined carbon ion particle therapy

**DOI:** 10.1007/s12032-025-03172-8

**Published:** 2025-12-24

**Authors:** Birgit Lohberger, Dietmar Glänzer, Vanessa Etschmaier, Slave Trajanoski, Andreas Leithner, Beate Rinner, Dietmar Georg

**Affiliations:** 1 Department of Orthopedics and Trauma, Medical University of Graz, Graz, Austria; 2 Core Facility Computational Bioanalytics, Medical University of Graz, Graz, Austria; 3 Division of Biomedical Research, Medical University of Graz, Graz, Austria; 4 Department of Radiation Oncology, Medical University of Vienna, Vienna, Austria; 5 MedAustron-Ion Therapy Center, Wiener Neustadt, Austria

**Keywords:** Sacral chordoma, Carbon ions irradiation, Crizotinib, DNA repair mechanisms

## Abstract

Chordoma resection is challenging due to proximity to the brainstem or spinal cord, and chemotherapy offers limited efficacy. Combining surgery with radiotherapy, particularly using carbon ions (C-ions) for their higher biological effectiveness, improves local control and survival rates. To investigate cellular mechanisms, two human sacral chordoma cell lines were irradiated with varying C-ions doses. Growth, cell cycle, DNA damage response, and protein phosphorylation were analyzed using flow cytometry, protein, and gene expression profiling. The potential of combining treatment with the ALK/MET inhibitor crizotinib to enhance radiosensitivity was also evaluated. C-ions irradiation resulted in a slight dose-dependent decrease in proliferation, a clear G_2_/M cell cycle arrest, and a significant activation of key regulators involved in DNA repair and damage response. The ALK/MET inhibitor crizotinib, considered a potential treatment for chordomas, reduced proliferation markers and modulated important genes related to DNA repair and cell cycle regulation, with *CDC20* and *FOXO4* being particularly significant. The phosphorylation of key regulators involved in DNA repair and damage prevention, as well as MAPKs activated by C-ions irradiation, was partially inhibited by the combination treatment with crizotinib. While crizotinib shows promise as a therapeutic agent for sacral chordomas, its capacity to enhance radiosensitivity appears limited.

**Supplementary Information:**

The online version contains supplementary material available at 10.1007/s12032-025-03172-8.

## Introduction

Chordoma is a rare, low-grade malignant tumor arising from notochordal remnants, typically located in the skull base, spine, or sacrum, with an incidence of < 1 in 1,000,000. It is classified into classic, chondroid, and dedifferentiated subtypes, with histological grading significantly impacting prognosis. The dedifferentiated type is particularly aggressive, with reduced 3-year survival rates [1]. Surgical resection remains the primary treatment for spinal and sacral chordomas but is associated with high morbidity and impaired mobility, affecting quality of life [2, 3]. Wide or marginal resection is achieved in 35–75% of sacral cases but only ~ 21% in spinal tumors due to frequent epidural involvement [4]. Combined surgery and radiotherapy may improve local control and survival outcomes [5, 6].

Chordoma are relatively radioresistant with a high rate of local recurrence and the potential for metastasis [7]. Therefore, high-dose, high-precision radiotherapy, as can be carried out using particle therapy with protons and carbon-ions (C-ions) is an important local treatment strategy [8–10]. Outcomes of protons and C-ions treatment of chordomas seem similar regarding tumor control, survival, and toxicity [11]. The higher relative biological effectiveness (RBE) of C-ions can improve treatment outcome compared to proton and photon radiotherapy. Despite the success in clinical application, very little is still known about the underlying cellular processes of C-ions therapy. In addition to radiotherapy, radiosensitizers have to potential to improve chordoma treatments. For example, ALK/MET inhibitors affected the growth behaviour and cMET phosphorylation state of chordoma cells in different ways and works more efficiently in sacral cells than in clival cells [12]. A drug screening study showed promising results for a combination with the EGFR inhibitor afatinib [13]. Crizotinib is an antineoplastic drug from the group of tyrosine kinase inhibitors that inhibits the receptor tyrosine kinases ALK, cMET, and ROS1. It is used for the treatment of non-small cell lung cancer [14]. Although chordomas exhibit considerable genetic and phenotypic variability, chromosome 7q31.2, which harbours the cMET receptor, frequently shows gains [15] whereby, MET signalling subsequently controls the survival, migration, and proliferation [16].

To address the above-mentioned knowledge gap, in the present study two human chordoma cell lines were irradiated with different doses of C-ions, and fundamental cellular processes such as growth, cell cycle, DNA damage response, and protein phosphorylation were examined. Additionally, the potential of a combined treatment with the ALK/MET inhibitor crizotinib to enhance radiosensitivity was investigated by studying growth behaviour, cell cycle distribution, apoptotic induction, and the expression of key DNA repair genes.

## Materials and methods

### Cell culture

The sacral chordoma cell lines MUG-Chor1 (CellBank MedUni Graz) [17] and U-CH2 (ATCC^®^ CRL-3218™, LGC Standards, Middlesex, UK) were cultured in IMDM/RPMI 4:1 (Life Technologies, Carlsbad, CA, USA) containing 10% fetal bovine serum, 1% insulin, transferrin, sodium selenite, 2 mM glutamine, and 1% penicillin/streptomycin (all Life Technologies). Incubation was carried out at 37 °C in a humidified atmosphere of 5% CO_2_. For C-ions irradiation experiments, adherent chordoma cells were plated in a density of 1 × 10^5^ cells/Nunc™ Lab-Tek™ flask on slides 9 cm^2^ (Thermo Fisher Scientific, Waltham, MA, USA) respectively 5 × 10^5^ cells/T25 flasks (Corning^®^, Merck KGaA, Darmstadt, Germany) and incubated overnight at 37 °C in a 5% CO_2_ environment to allow the cells to settle.

### Experimental C-ions irradiation conditions

C-ion irradiation was conducted at MedAustron (Wiener Neustadt, Austria) [18] using a horizontal beam line with active spot scanning. Samples embedded in a water phantom were positioned via a precision robot couch and laser-guided to the isocenter. Treatment plans ensuring homogeneous dose coverage (17 × 9 cm² field) were generated with RayStation TPS (RaySearch Laboratories, Stockholm, Sweden). The treatment plan featured a 4 cm spread-out Bragg peak length (SOBP; 6–10 cm depth) using 1–2 mm energy layer spacing and energies from 170 to 239 MeV/u. Dose-averaged dose averaged linear energy transfer (LET_d_) along the SOBP ranged from 13 to 206 keV/µm. In the central SOBP region where cells were placed, positioning uncertainties (± 0.5 mm) had minimal impact, yielding a LET_d_ of 56 ± 1 keV/µm. LET_d_ values were obtained via Monte Carlo simulations using GATE (Geant4), and the physical accuracy of the setup has been validated in prior studies [19, 20].

### xCELLigence^®^ proliferation analysis

Real-time cell proliferation was monitored every 20 min over 96 h using the xCELLigence^®^ system with E-Plates^®^ (OLS, Bremen, Germany). Impedance-based cell index values, reflecting cell number and activity, were recorded and analyzed with RTCA 2.0 software (OLS).

### Viability assay

Cell viability was assessed using the CellTiter-Glo^®^ assay (Promega) and normalized to untreated controls. Background was subtracted using culture media. Luminescence was measured at 375 nm with a LUMIstar™ luminometer (BMG Labtech) (mean ± SD; *n* = 3; biological quadruplicates). Cells were treated with 5 µM crizotinib (Selleckchem Houston, TX, USA; 10 mM DMSO stock) for 24 h before 4 Gy C-ions irradiation.

### Flow cytometry cell cycle analysis

Cells were pre-treated with 5 µM crizotinib for 24 h, irradiated with 4 Gy C-ions, and incubated for another 48 h with the inhibitor. After 72 h total, cells were harvested by trypsinization, fixed in 70% ice-cold ethanol (10 min, 4 °C), washed, and stained with propidium iodide (PI)-staining buffer (50 µg/mL PI, 100 µg/mL RNase A, 0.1% sodium citrate, 0.1% Triton X-100) for 15 min at 37 °C. Cell cycle analysis was performed using CytoFlexLX (Beckman Coulter, Pasadena, CA, USA) and ModFit LT v4.1.7 (Verity software house). All experiments were conducted in five independent biological replicates.

### Protein expression analysis

For immunoblotting, whole-cell protein extracts were prepared with a lysis buffer (50 mM Tris-HCl pH 7.4, 150 mM NaCl, 1 mM NaF, 1 mM EDTA, 1% NP-40, 1 mM Na3VO4) and a protease inhibitor cocktail (P8340; Sigma Aldrich, St. Louis, MI, USA), 1 h and 48 h after C-ions irradiation (which corresponds to a total incubation time of crizotinib of 24 h and 72 h).

The following groups were analyzed: untreated controls (ctrl 0 Gy), and cells irradiated with 2, 4, or 8 Gy C-ions; for crizotinib blots: 5 µM crizotinib alone (Crizo 0 Gy) and combined with 4 Gy C-ions (Crizo 4 Gy). Protein concentrations were measured using the Pierce BCA Assay (Thermo Fisher). Proteins were separated by SDS-PAGE and transferred to Amersham™ Protran™ 0.45 μm nitrocellulose membranes (GE Healthcare Life Science, Little Chalfont, UK). Western blotting was performed using primary antibodies (1:1000) against apoptotic markers (cleaved caspase 8/9, cleaved PARP, survivin), DNA damage markers (γH2AX, p-DNA-PKcs, p-ATM, p-CHK1/2, p-p53), and MAPKs (p-AKT/AKT, p-ERK/ERK, p-p38/p38), all from Cell Signaling. β-actin (1:10000, Santa Cruz) served as loading control. HRP-conjugated secondary antibodies (Dako) and Amersham™ ECL™ Prime were used for detection. Signals were captured with the ChemiDocTouch system and analyzed using ImageLab 5.2 (BioRad). Original membranes are shown in Figure S1.

### Gene expression profiling

Gene expression profiling was performed using the Thermo Fisher Ion AmpliSeq RNA workflow. RNA was extracted with the RNeasy Mini Kit (Qiagen, Hilden, Germany) and reverse transcribed using the SuperScript™ VILO™ Kit. cDNA (from 50 ng RNA) was amplified with a custom Ion AmpliSeq RNA Panel targeting 69 genes. Libraries were prepared using the AmpliSeq Library Kit Plus and quantified with the Ion Library TaqMan™ Kit. Sequencing was done on an Ion S5XL system using a 540 Chip and 200 bp workflow, achieving ~ 1 million reads per sample. Gene expression was quantified as reads per million using the AmpliSeq RNA Ion Torrent Suite Plugin (v4.4.0.4). For each of the 8 samples, mapped reads, percent on-target, and percent assigned reads were reported.

### Reverse transcription polymerase chain reaction (RT-PCR)

Total RNA was isolated 72 h after combined treatment with 5 µM crizotinib and 4 Gy C-ion IR using the RNeasy Mini Kit with DNase-I treatment (Qiagen, Hilden, Germany). 2 µg RNA were reverse transcribed using the iScript cDNA Synthesis Kit (Bio-Rad) with oligo(dT) and random primers. qPCR was performed in technical triplicates using SYBR Green Supermix and measured on the CFX96 Touch system (Bio-Rad). The following QuantiTect primer assays (Qiagen) were used: *cMyc*, survivin (*BIRC5*), *PCNA* (proliferation) and *BRCA1*,* SMC1A*,* FANCD2*,* CREBBP*,* cdc25c*,* MDM2* (DNA damage response). The results were analyzed using the CFX manager software for CFX Real-Time PCR Instruments (Bio-Rad; version 3.1). Ct values > 32 were excluded. Relative expression was calculated using the ∆∆Ct method, based on the geometric mean of the reference genes RPL and TBP. Target gene Ct values were normalized to reference genes (ΔCt), and sample ΔCt values to control ΔCt (ΔΔCt). Expression ratios were determined using the 2^−ΔΔCt^ method (*n* = 8; biological triplicates).

### Statistical analysis

Statistical analyses were performed using IBM SPSS Statistic 29.0.0.0 (241) (New York, NY, USA), and graphical representation was performed using the SigmaPlot 14.5 software (SYSTAT, Palo Alto, CA, USA). Data were tested for normality with the Kolmogorov–Smirnov test. Since data distribution in all the samples significantly deviated from the normal distribution, the statistical significance of the observed differences was tested with non-parametric tests. Single comparisons were tested using the Mann-Whitney U test. Multiple comparisons were tested with Kruskal–Wallis H test, followed by pairwise analysis with Bonferroni correction. *p*-values were considered statistically significant if they were less than 0.05 */#, 0.01 **/##, or 0.001 ***/###, indicating the level of significance. Read counts from the gene expression profiling were further processed and analysed for statistically significant changes using statistical program R Version 4.3.3 [21] over the RStudio IDE [22] extended with the packages DESeq2 [23] and EnhancedVolcano [24]. Significantly deregulated genes were selected with the adjusted *p*-value less than 0.05 and log-fold change ratio more than 1.

## Results

### Response of sacral chordoma cells to C-ions irradiation

**Fig. 1:**
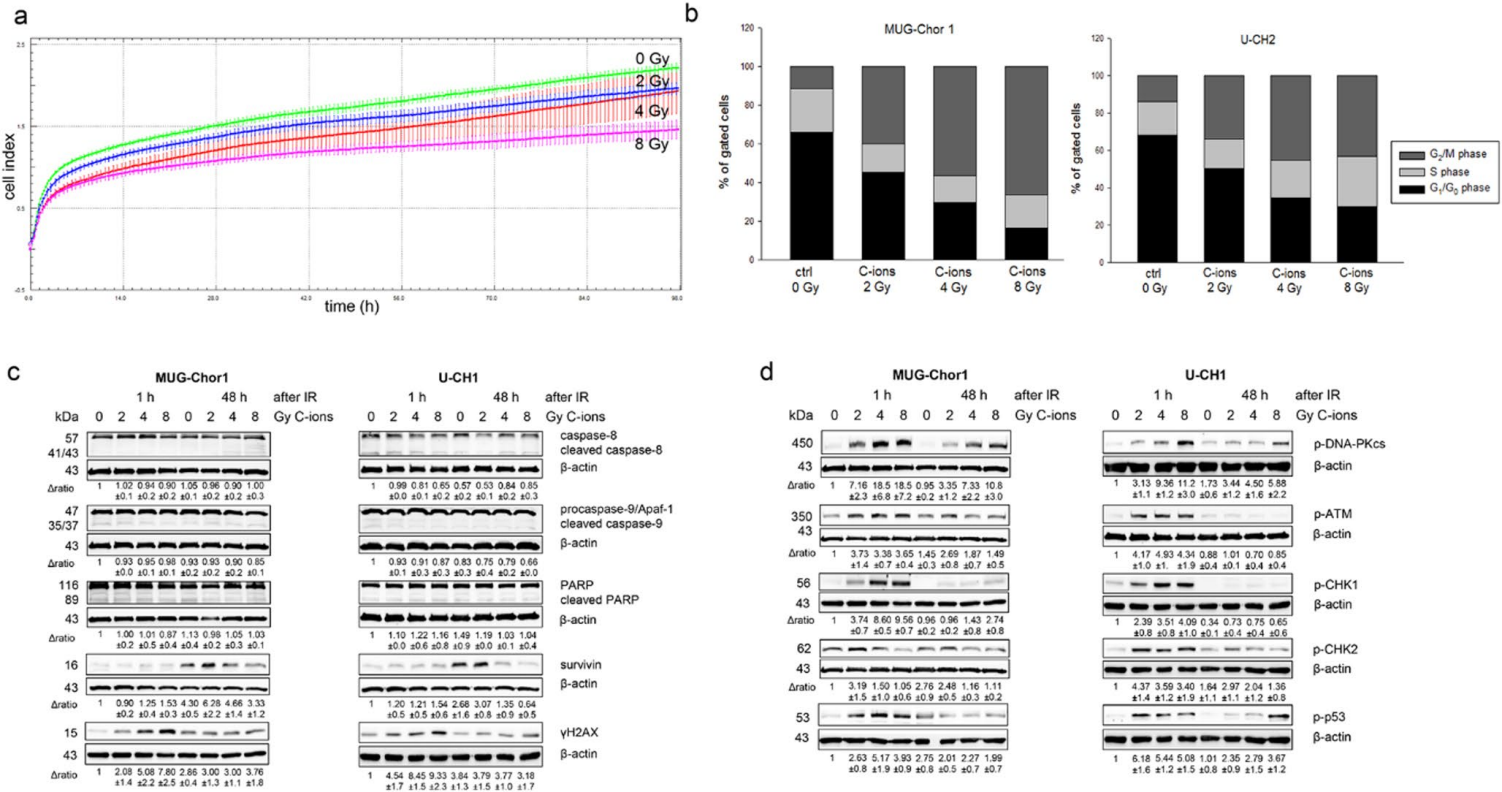
Proliferation analysis, cell cycle distribution, apoptotic induction and protein phosphorylation after C-ions irradiation. (**a**) The xCELLigence real-time proliferation analysis revealed a moderate dose-dependent reduction in proliferation after C-ions irradiation (green: non-IR control cells; blue: 2 Gy; red: 4 Gy; magenta: 8 Gy) in MUG-Chor1 cells. (**b**) Cell cycle distribution was analyzed were by flow cytometry 48 h after C-ions irradiation. The corresponding statistical evaluation shown in stacked bar charts (*n* = 5). Both cell lines were clearly arrested in the G_2_/M phase. The influence of C-ions irradiation on (**c**) apoptotic induction and (**d**) protein phosphorylation of DNA-PKcs, ATM, CHK1, CHK2, and p53 was evaluated by immunoblotting under control conditions (0 Gy) and 1 and 48 h after 2, 4, and 8 Gy C-ions irradiation. β-actin was used as loading control

Using the xCELLigence real-time analysis, a moderate dose-dependent reduction in proliferation was found after C-ions IR (green: non-IR control cells; blue: 2 Gy; red: 4 Gy; magenta: 8 Gy) (Fig. 1a). A critical aspect of tumor biology involves the disruption of the cell cycle caused by therapeutic interventions. To assess cell cycle effects, flow cytometry was performed 48 h after C-ion irradiation. Irradiation led to a reduction in G_1_/G_0_ (black bars) and S phase cells (light grey bars), with a marked increase in G_2_/M phase cells (dark grey bars), indicating G_2_/M arrest in both cell lines (Fig. 1b). The corresponding mean ± SEM values and statistical analysis are listed in the Table S1.

To assess apoptosis, total protein was isolated 1 h and 48 h post-irradiation, and cleavage of caspase-8, caspase-9, and PARP was analyzed. Immunoblotting showed no caspase-dependent apoptosis in response to increasing C-ion doses. However, survivin expression decreased dose-dependently at 48 h, suggesting apoptotic suppression (Fig. 1c). A dose-dependent increase in γH2AX phosphorylation was detected 1 h post-IR, indicating DNA damage, which persisted at 48 h only at higher doses. To assess key DNA damage regulators, proteins were isolated from chordoma cells after 0, 2, 4, and 8 Gy C-ions. Phosphorylation changes were prominent at 1 h post-irradiation but minimal at 48 h. p-DNA-PKcs, p-ATM, p-CHK1, and p-p53 increased dose-dependently, while p-CHK2 decreased with higher irradiation doses (Fig. 1d). A representative blot (*n* = 3) is shown with β-actin as loading control. Fold changes (Δratio) relative to non-IR controls are shown as mean ± SD. Full blots are in Fig. S1.

### Regulation of important cell cycle and DNA repair genes under the influence of crizotinib

**Fig. 2:**
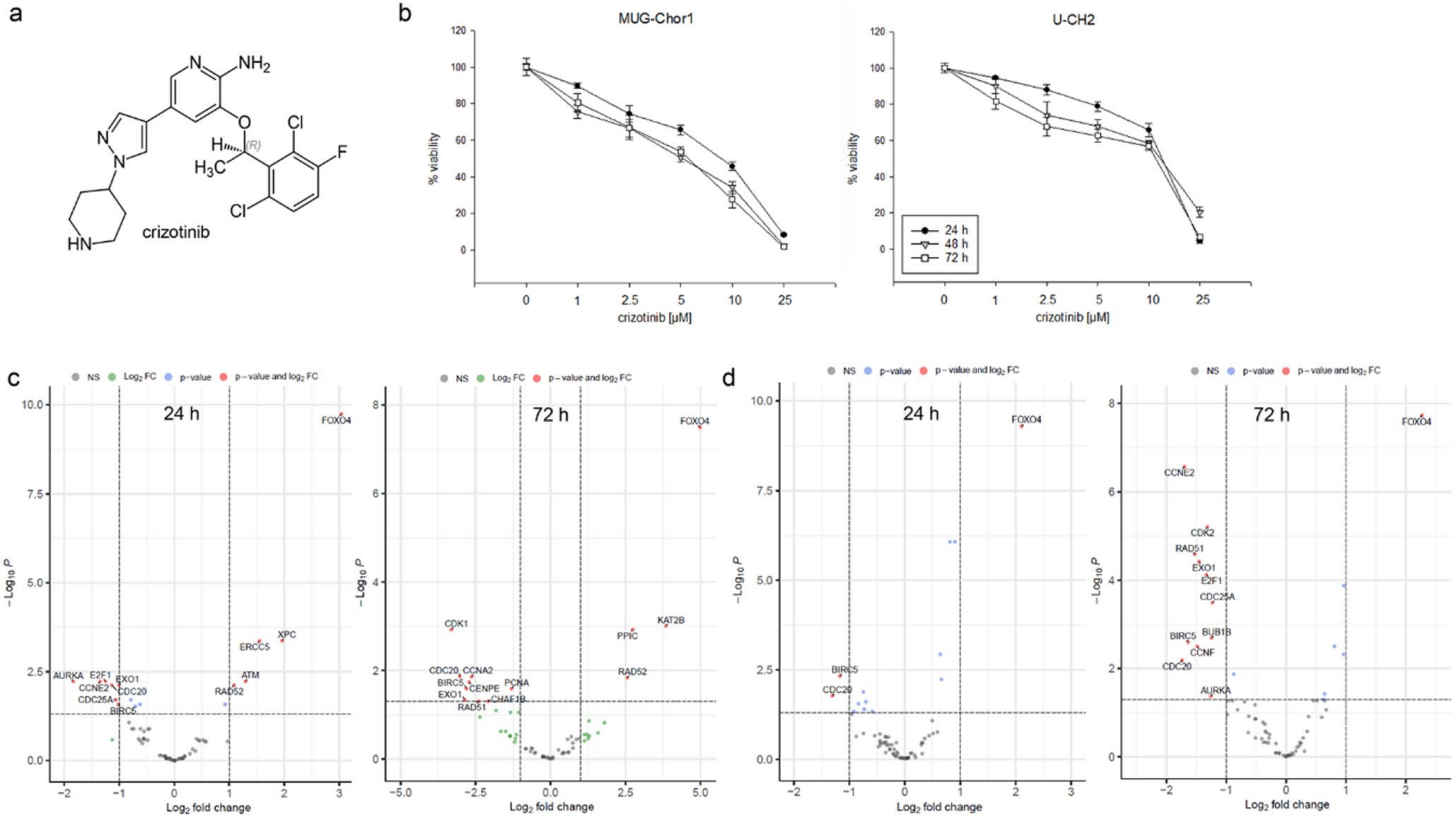
The cell biological impact of crizotinib. (**a**) Chemical structure of crizotinib. (**b**) The percentage of metabolic ATP levels, which is representative for the viability of the cells (mean ± SD; *n* = 3; measured in quadruplicates). Volcano blots of RNA sequencing data of relevant cell cycle and DNA repair regulator genes presented in log2 fold-change of (**c**) MUG-Chor1 and (**d**) U-CH2 cells 24 h and 72 h after 5 µM crizotinib treatment (*n* = 8)

The ALK/MET inhibitor crizotinib (Fig. 2a) affects sacral chordoma cell growth and cMET phosphorylation, making it a potential therapeutic candidate. Cells were treated with 0–25 µM crizotinib, and viability was measured at 24, 48, and 72 h, showing no significant time-dependent differences (Fig. 2b). IC₅₀ values at 72 h, determined using a non-linear regression with a four-parameter logistic model, were 4.57 µM for MUG-Chor1 and 6.79 µM for U-CH2. A concentration of 5 µM was used for all further experiments. Volcano plots of RNA sequencing data (Fig. 2c and d) show log₂ fold changes in cell cycle and DNA repair gene expression after 24 h and 72 h crizotinib treatment in MUG-Chor1 and U-CH2 cells. In the MUG-Chor1 cell lines significant downregulation of the proliferation markers *BIRC5* and *PCNA*, the mitotic cluster genes *CDC20* and *CENPE*, the chromatin assembly factor 1 (*CHAF1B*), the G_2_/M phase regulating cyclin dependent kinase 1 (*CDK1*) and cyclin A2 (*CCNA2*), the exonuclease 1 (*EXO1*) and *Rad51* can be observed compared to the untreated control. However, the senescence gene forehead box 04 (*FOXO4*), the G_1_/S DNA packaging gene *KAT2B*, *PPIC*, and the DNA repair protein *Rad52* were significantly upregulated. In the U-CH2 cell line *BIRC5*, *EXO1*, the mitotic cluster genes *cdc20* and *BUB1B*, the aurora kinase A (*AURKA*), the cell cycle control genes cyclin E2 (*CCNE2*), *CDC25a*,* Rad51*,* E2F1*,* CCNF*, and *CDK2* were significantly downregulated. *FOXO4* is also significantly upregulated in this cell line. Full log₂ fold change and p-values are listed in Table 1.

**Table 1:** Significant differences obtained by gene expression profiling between untreated control and MUG-Chor1 and U-CH2 cells treated with 5 µM Crizotinib for 24 h and 72 h. Log2 fold changes (log2 FC) and their corresponding p-values are listed (*n* = 8)

	24 h			72 h		
	**gene**	**log2 FC**	**p-value**	**gene**	**log2 FC**	**p-value**
MUG-Chor1	*ATM*	1.294	0.0005	*BIRC5*	−2.8052	0.0038
	*AURKA*	−1.837	0.0004	*CCNA2*	−2.6323	0.0013
	*BIRC5*	−1.019	0.0059	*CDC20*	−3.0262	0.0011
	*CCNE2*	−1.348	0.0005	*CDK1*	−3.3092	7.54E-05
	*CDC20*	−1.138	0.0010	*CENPE*	−2.7164	0.0023
	*CDC25A*	−1.062	0.0032	*CHAF1B*	−2.0861	0.0102
	*E2F1*	−1.250	0.0006	*EXO1*	−2.8633	0.0081
	*ERCC5*	1.548	1.94E-05	*FOXO4*	4.9865	5.04E-10
	*EXO1*	−1.006	0.0011	*KAT2B*	3.8506	3.12E-05
	*FOXO4*	3.038	2.74E-12	*PCNA*	−1.3000	0.0040
	*RAD52*	1.087	0.0011	*PPIC*	2.7403	7.45E-05
	*XPC*	1.959	1.28E-05	*RAD51*	−2.4031	0.0096
				*RAD52*	2.5604	0.0016
U-CH2	*BIRC5*	−1.171	0.0003	*AURKA*	−1.259	0.0106
	*CDC20*	−1.304	0.0019	*BIRC5*	−1.636	0.0003
	*FOXO4*	2.107	7.40E-12	*BUB1B*	−1.246	0.0002
				*CCNE2*	−1.698	7.81E-09
				*CCNF*	−1.486	0.0005
				*CDC20*	−1.738	0.0013
				*CDC25A*	−1.232	3.82E-05
				*CDK2*	−1.314	2.73E-07
				*E2F1*	−1.332	6.61E-06
				*EXO1*	−1.457	2.77E-06
				*FOXO4*	2.271	2.73E-10
				*RAD51*	−1.524	1.53E-06

### Impact of a combined treatment of crizotinib and C-ions irradiation

**Fig. 3:**
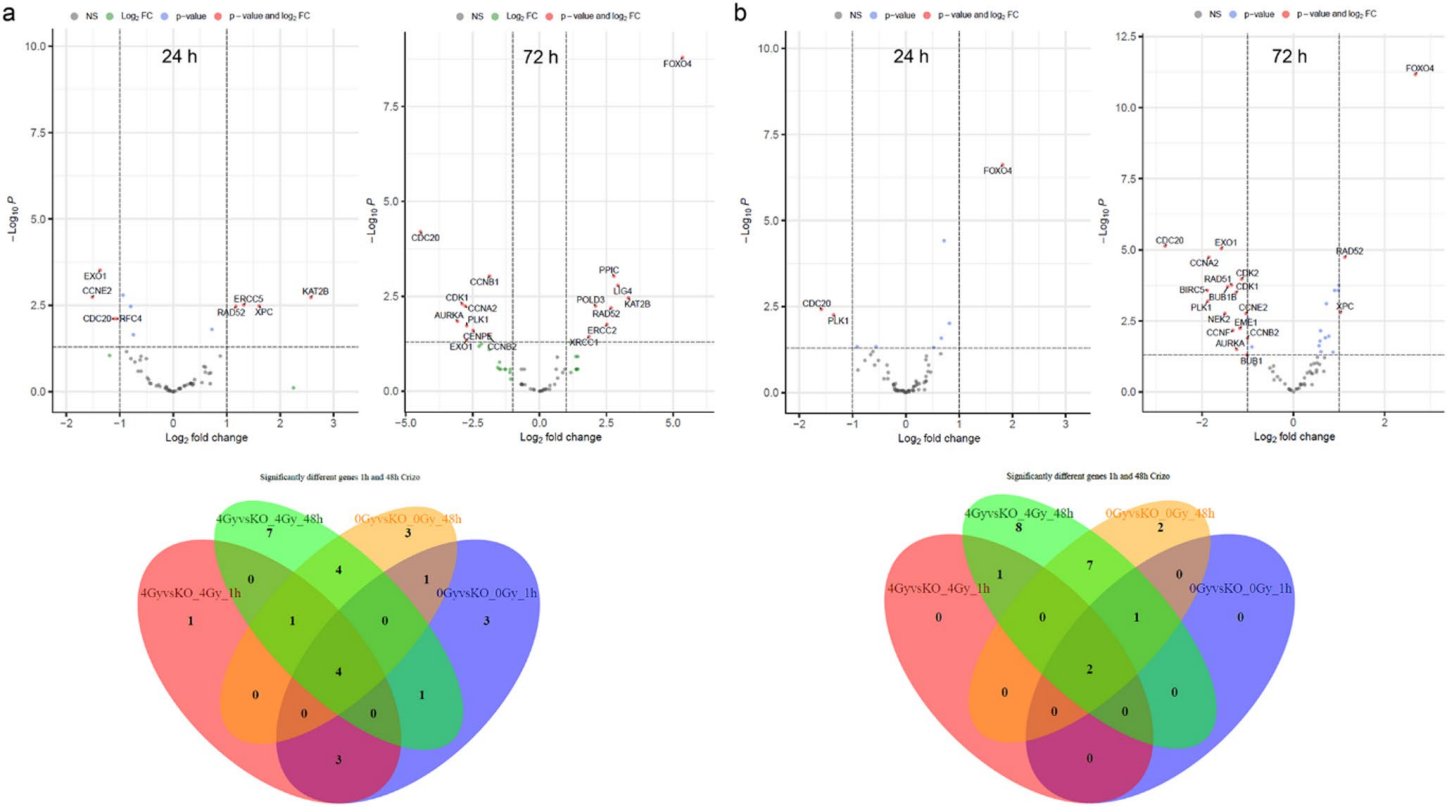
Impact of combined treatment of crizotinib and C-ion irradiation in gene expression. Volcano blots of RNA sequencing data of relevant cell cycle and DNA repair regulator genes presented in log2 fold-change of (**a**) MUG-Chor1 and (**b**) U-CH2 cells 24 h and 72 h after combined treatment with 5 µM crizotinib treatment and 4 Gy C-ions irradiation (*n* = 8). The corresponding Venn diagrams illustrate the intersections and differences between the various groups. The untreated controls are labelled KO

To assess whether crizotinib sensitizes chordoma cells to C-ions radiotherapy, cells were pre-treated with crizotinib for 24 h prior to 4 Gy C-ions irradiation. Gene expression profiling showed a significant upregulation of key DNA repair genes following combined treatment, such as *ERCC2*, *LIG4*, *Rad52*, and *XRCC1*, as well as the cell cycle regulators *KAT2B*, *POLD3*, *PPIC*, and the senescence marker *FOXO4* in MUG-Chor1 (Fig. 3a). However, *AURKA*, the cyclins *CCNA2*, *CCNB1*, *CCNB2*, and *CDC20*, *CDK1*, *EXO1*, and *CENPE* were significantly reduced. U-CH2 showed a very similar result (Fig. 3b). The corresponding Venn blots show the intersections and differences between the various groups. *CDC20*, *FOXO4*, *EXO1*, and *Rad52* in MUG-Chor1 and *CDC20* and *FOXO4* in U-CH2 were found to be the central significantly altered genes. All corresponding log2 values and p-values are provided in Table 2.

**Table 2:** Significant differences obtained by gene expression profiling between C-ions irradiated MUG-Chor1 and U-CH2 cells and 1 h and 48 h after after combined treatment with 5µM Crizotinib and 4 Gy C-ions. Log2 fold changes (log2 FC) and their corresponding p-values are listed (*n* = 8)

	24 h			72 h		
	**gene**	**log2 FC**	**p-value**	**gene**	**log2 FC**	**p-value**
MUG-Chor1	*CCNE2*	−1.515	0.0001	*AURKA*	−3.069	0.0024
	*CDC20*	−1.112	0.0013	*CCNA2*	−2.780	0.0006
	*ERCC5*	1.318	0.0002	*CCNB1*	−1.870	5.51E-05
	*EXO1*	−1.369	9.03E-06	*CCNB2*	−1.908	0.0076
	*FOXO4*	3.269	5.62E-14	*CDC20*	−4.452	1.95E-06
	*KAT2B*	2.575	0.0001	*CDK1*	−2.894	0.0005
	*RAD52*	1.172	0.0004	*CENPE*	−2.484	0.0053
	*RFC4*	−1.056	0.0013	*ERCC2*	2.520	0.0032
	*XPC*	1.608	0.0003	*EXO1*	−2.724	0.0117
				*FOXO4*	5.348	2.51E-11
				*KAT2B*	3.325	0.0003
				*LIG4*	2.929	0.0001
				*PLK1*	−2.722	0.0037
				*POLD3*	2.094	0.0007
				*PPIC*	2.784	5.71E-05
				*RAD52*	2.678	0.0009
				*XRCC1*	1.850	0.0089
U-CH2	*CDC20*	−1.586	0.0001	*AURKA*	−1.241	0.0135
	*FOXO4*	1.815	3.51E-09	*BIRC5*	−1.887	4.05E-05
	*PLK1*	−1.357	0.0003	*BUB1*	−1.021	0.0234
				*BUB1B*	−1.438	2.63E-05
				*CCNA2*	−1.851	1.34E-06
				*CCNB2*	−1.007	0.0048
				*CCNE2*	−1.038	0.0004
				*CCNF*	−1.325	0.0021
				*CDC20*	−2.810	2.18E-07
				*CDK1*	−1.252	6.06E-05
				*CDK2*	−1.132	9.46E-06
				*EME1*	−1.169	0.0017
				*EXO1*	−1.577	3.97E-07
				*FOXO4*	2.670	9.92E-14
				*NEK2*	−1.500	0.0004
				*PLK1*	−1.875	0.0001
				*RAD51*	−1.353	1.96E-05
				*RAD52*	1.128	1.23E-06
				*XPC*	1.029	0.0003

**Fig. 4:**
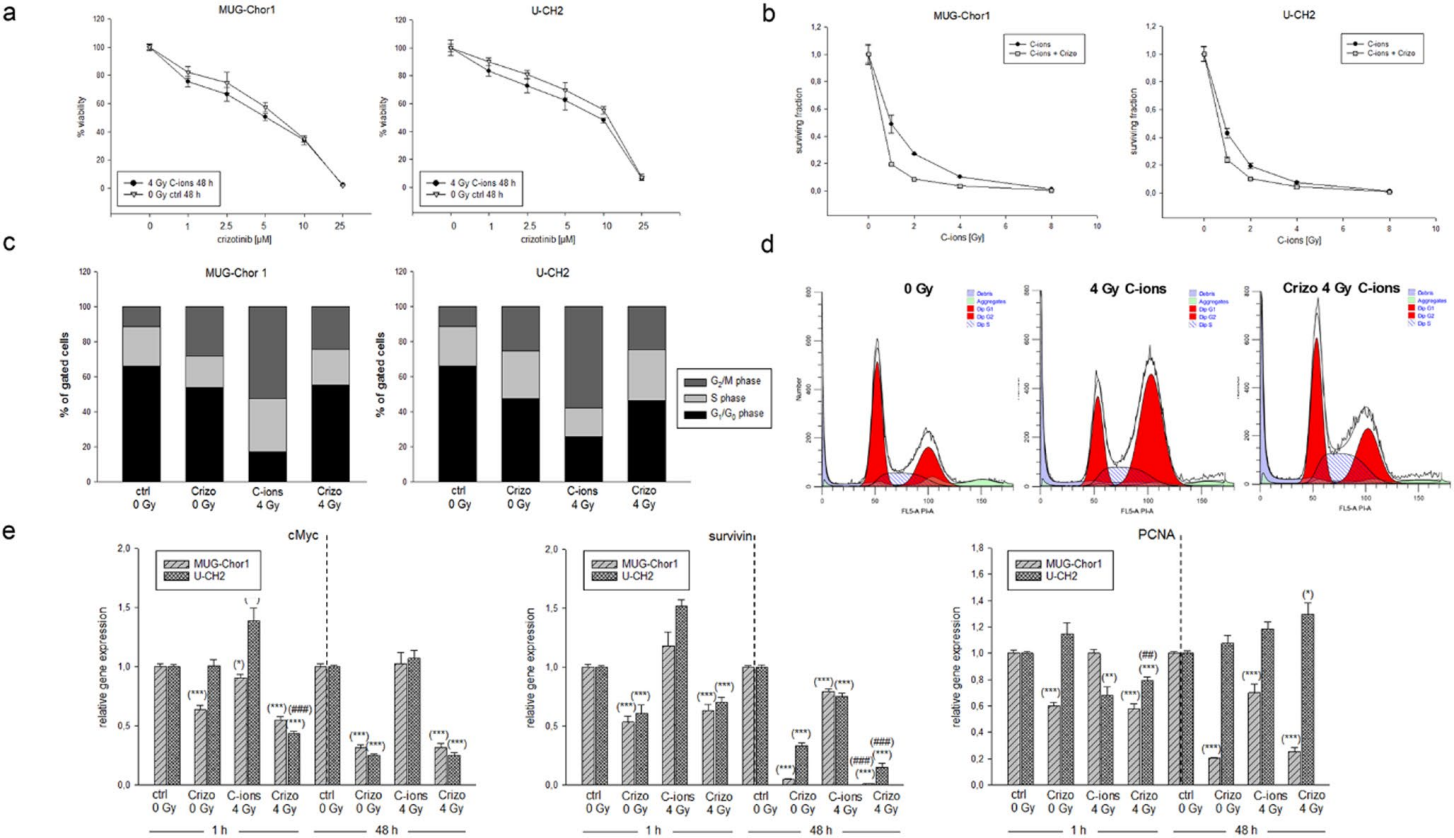
The impact of the combined treatment of crizotinib and C-ions irradiation on viability, clonogenic survival, cell cycle, and proliferation markers. (**a**) Viability analysis based on metabolic ATP levels revealed minor differences between the non-irradiated controls and 4 Gy C-ions irradiation. (b) For clonogenic survival assay (CFU) cells were harvested immediately after 0, 1, 2, 4, and 8 Gy C-ions irradiation and seeded according the dose level. Colonies were stained with crystal violet and the surviving fractions were analysed. C-ion irradiation, repectively combined treatment with crizotinib, inhibited the ability of sacral chordoma cells to form colonies in a dose-dependent manner (mean ± SD; *n* = 4; measured in 6-fold determination). (**c**) Cells were treated with 5 µM crizotinib alone (Crizo 0 Gy), irradiated with 4 Gy C-ions (C-ions 4 Gy), or subjected to the combined treatment (Crizo 4 Gy). The statistical evaluation is presented in stacked bar charts (*n* = 5) and (**d**) representative measurements are shown. (**e**) Gene expression analysis of proliferation-related genes cMyc, *PCNA*, and survivin (*BIRC5*) were evaluated using RT-qPCR. MUG-Chor1 (light grey striped) and U-CH2 (dark grey dotted) chordoma cells were treated with 5 µM crizotinib alone or in combination with 4 Gy C-ions irradiation (mean ± SD, *n* = 8, measured in triplicates). Statistical significances compared to the untreated controls (ctrl 0 Gy) are defined as follows: * *p* < 0.05; ** *p* < 0.01; *** *p* < 0.001. Statistical significances between the 5 µM crizotinib group and the combined treatment with C-ions irradiation are indicated as # *p* < 0.05; ## *p* < 0.01; ### *p* < 0.001

Viability analysis based on metabolic ATP measurements showed only minor differences between non-irradiated controls and cells exposed to 4 Gy C-ions (Fig. 4a). In the clonogenic survival assay (CFU), cells were collected immediately after exposure to 0, 1, 2, 4, or 8 Gy C-ions and seeded according to dose. Following crystal violet staining, surviving fractions were quantified. C-ions irradiation, either alone or in combination with crizotinib, reduced the clonogenic capacity of sacral chordoma cells in a dose-dependent manner (Fig. 4b). Flow cytometry analysis confirmed the G_2_/M arrest after 4 Gy C-ions irradiation. Notably, combined treatment with crizotinib appeared to reverse this arrest, with cell cycle distribution resembling that of untreated control cells (Fig. 4c). Representative blots are shown for visualization (Fig. 4d). Mean ± SEM values and significance are provided in Table S2.

To examine the regulation of key proliferation-related genes (*cMyc*,* PCNA*) and the survival marker survivin (*BIRC5*) following combined treatment, we performed RT-qPCR analysis on RNA isolated 48 h after irradiation. Given the 24 h pre-treatment, this corresponds to 72 h of total crizotinib exposure. We analyzed cells treated with 5 µM crizotinib alone (Crizo 0 Gy), irradiated with 4 Gy C-ions (C-ions 4 Gy), or combined treatment (Crizo 4 Gy) (Fig. 4e). Crizotinib alone significantly reduced the expression of *cMyc*, *PCNA*, and survivin both immediately and 48 h post-irradiation. In contrast, C-ions alone had little effect. Combined treatment further significantly reduced *cMyc* and survivin expression.

**Fig. 5:**
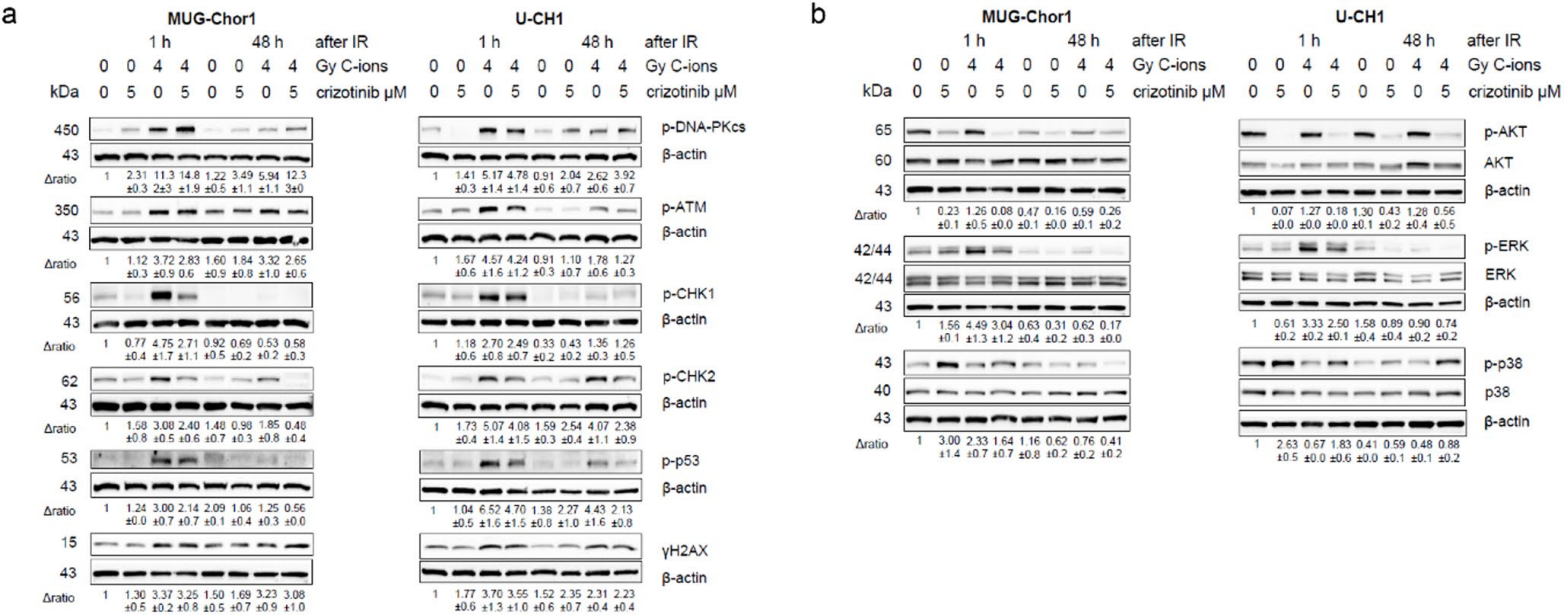
DNA repair regulators and expression of downstream genes after the combined treatment. (**a**) Protein phosphorylation of DNA-PKcs, ATM, CHK1, CHK2, and p53 and (**b**) the MAPKs p-AKT, p-ERK, and p-p38 was evaluated by immunoblotting under control conditions (ctrl; 0 Gy) and 1 and 48 h after 4 Gy C-ions irradiation, respectively in combination with 5 µM crizotinib. β-actin was used as loading control

To further investigate the DNA damage response following combined treatment, total protein was isolated 1 h and 48 h after 4 Gy C-ions irradiation and analyzed by immunoblotting (Fig. 5a). Crizotinib treatment alone did not affect phosphorylation, whereas 4 Gy C-ions significantly increased p-DNA-PKcs, p-ATM, p-CHK1, p-CHK2, p-p53, and the DNA damage marker γH2AX expression. Due to rapid phosphorylation, these effects were more prominent 1 h after IR. Combined treatment with crizotinib reduced phosphorylation compared to irradiation alone, correlating with cell cycle restoration. Additionally, crizotinib treatment decreased AKT and ERK phosphorylation induced by irradiation, while p38 activation was increased (Fig. 5b). A representative blot (*n* = 3) is shown, with β-actin as a loading control. Δratio values, normalized to non-IR controls, are presented as mean ± SD. Full blots are in Fig. S1.

**Fig. 6:**
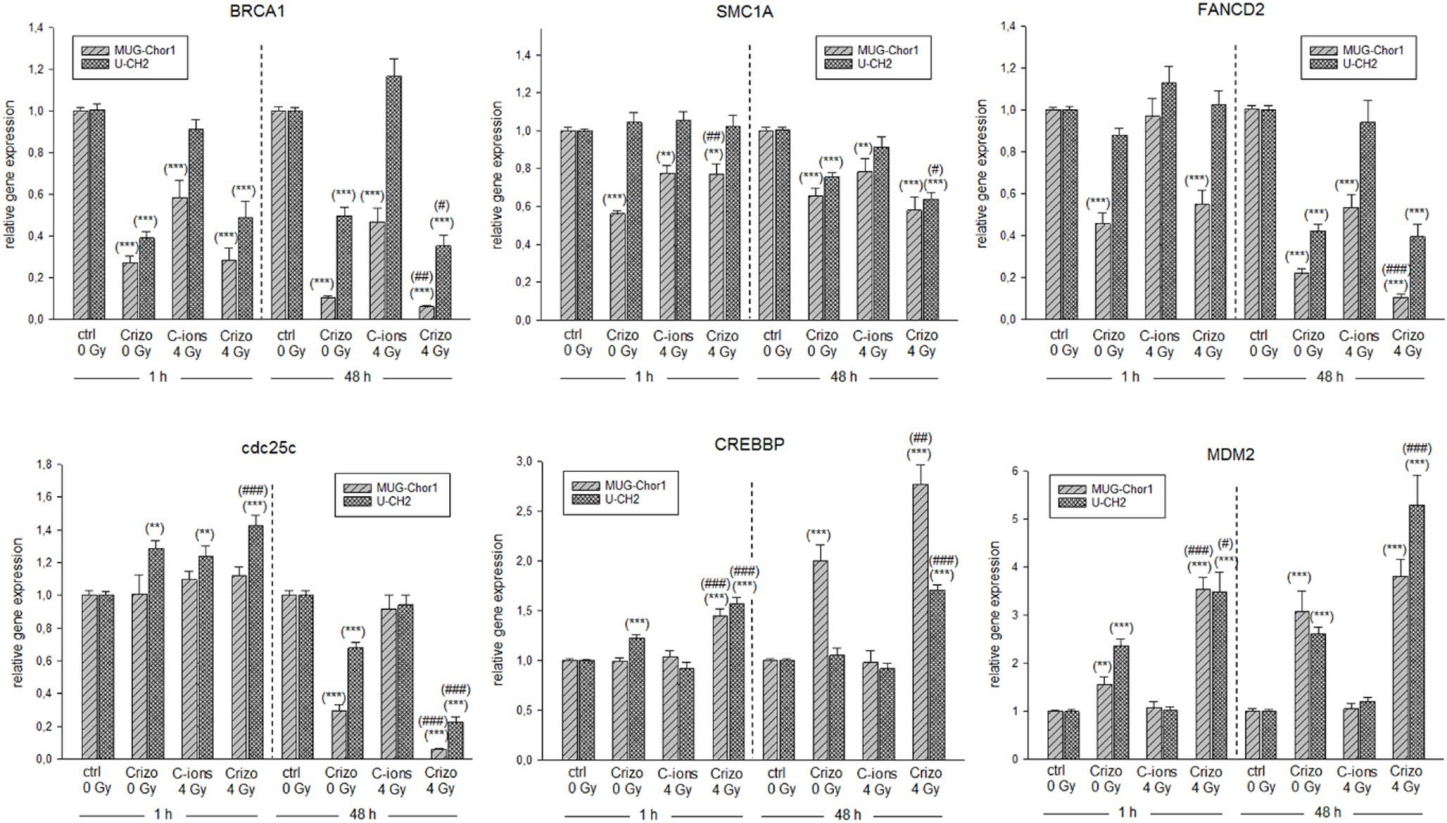
The effect of the combined treatment of crizotinib and C-ions irradiation on the expression of DNA repair and damage prevention key genes. MUG-Chor1 (light grey striped) and U-CH2 (dark grey dotted) chordoma cells were treated with 5 µM crizotinib alone or in combination with 4 Gy C-ions irradiation (mean ± SD, *n* = 8, measured in triplicates). Statistical significances compared to the untreated controls (ctrl 0 Gy) are defined as follows: * *p* < 0.05; ** *p* < 0.01; *** *p* < 0.001. Statistical significances between the 5 µM crizotinib group and the combined treatment with C-ions irradiation are indicated as # *p* < 0.05; ## *p* < 0.01; ### *p* < 0.001

Relative gene expression analysis of the DNA damage key genes was performed using RT-qPCR (Fig. 6). The same groups were analysed as described above. Crizotinib treatment significantly reduced the expression of ATM kinase downstream targets *BRCA1*, *SMC1A* and *FANCD2* both 1 h and 48 h after IR, while this effect was only observed with *cdc25c* after 48 h. C-ions irradiation alone had minimal impact on gene expression. However, combined treatment with 5 µM crizotinib and 4 Gy C-ions (Crizo 4 Gy) led to a significant downregulation of these genes. *CREBBP* expression increased significantly over time. Gene expression of the AKT downstream target *MDM2* also significantly increased following combined treatment, likely due to a negative feedback loop from reduced AKT phosphorylation.

## Discussion

Despite its clinical application, robust research on the fundamental cellular mechanisms in sacral chordoma cells following C-ions therapy remains limited in the literature, especially considering the complexities associated with this challenging therapeutic context. Our work aims to initiate efforts to address this gap. Initial basic data from sacral chordoma cells exposed to C-ions IR at doses of 2, 4, and 8 Gy indicated a slight dose-dependent reduction in proliferation, along with a clear arrest of the cells in the G_2_/M phase of the cell cycle. These observations are largely consistent with findings in other malignant bone tumors, such as chondrosarcoma [25, 26]. Furthermore, no apoptotic induction was observed in our cellular model, along with a reduction in the survival marker survivin (*BIRC5*) and a clear dose-dependent expression of the DNA damage marker γH2AX. Zhang et al. demonstrated, that high-doses of ionizing radiation significantly reduced cell viability and colony formation rate of U-CH1 and U-CH2 chordoma cell lines, whereas the effects of low-dose radiation were insignificant [27]. Although C-ions irradiation induces only a modest, dose-dependent reduction in overall cell proliferation, the clonogenic potential of the cells is markedly diminished. This effect is further amplified when combined with crizotinib, suggesting that while many cells remain metabolically active, their capacity for long-term survival and colony formation is more severely compromised, potentially due to DNA damage, impaired repair mechanisms, or inhibition of critical survival pathways. Irradiation with 2, 4, and 8 Gy of C-ions dose-dependently activated the key regulators of all major DNA damage response and repair pathways, such as DNA-PKcs, ATM, CHK1, CHK2, and p53, through phosphorylation.

A comprehensive genomic and transcriptomic analyses identified the IGF1R/FGFR/EGFR and CDK4/6 pathways as treatment strategies for chordoma patients [28]. Furthermore, a screening of 133 approved cancer drugs, which included tyrosine kinase inhibitors, HDAC inhibitors, and proteasome inhibitors, the ALK/MET inhibitor crizotinib demonstrated promising potential for standard chemotherapy regimens for chordomas [13]. Immunohistochemical staining on chordoma patients tissue samples as well as on different chordoma cell lines showed a moderate or strong expression of membrane and cytoplasmic cMET. Crizotinib inhibited chordoma cell growth, induced apoptosis and cell cycle arrest, and suppressed downstream pathways such as AKT, ERK, and STAT3 [12]. Based on this initial data, we aimed to investigate the potential of a combined therapy of crizotinib and C-ions irradiation. A desirable effect would be sensitization to radiotherapy through the inhibition of DNA repair pathways and modulation of the DNA damage response. According to the dose-response curves, we chose a concentration of 5 µM crizotinib for our in vitro studies. Using RNA sequencing, over 70 selected regulatory genes of the cell cycle and DNA repair pathways were examined following crizotinib treatment.

The activation of cell cycle checkpoints and DNA repair pathways is critical for maintaining genomic stability. Genotoxic damage triggers a complex network of interconnected DNA damage checkpoints and repair mechanisms. Post-translational modifications of various proteins involved in both checkpoint activation and DNA repair are essential for orchestrating this cellular response. Throughout cell division, multiple cell cycle checkpoints are in place to ensure the accuracy of cellular processes, allowing the cycle to be paused at nearly any transition point if significant errors or DNA damage are detected [29]. In response to DNA damage kinases ATM and ATR phosphorylate target proteins on serine and threonine residues, thereby activating the DNA damage checkpoint. The effector kinases CHK1 and CHK2 play a crucial role in amplifying the checkpoint signal across the cell. Once activated, CHK1 and CHK2 phosphorylate cdc25a during the G_1_/S-phase transition and cdc25c during the G_2_/M transition, effectively halting cell cycle progression [30]. Both after 24 h and 72 h, the majority of the significantly altered DNA repair and cell cycle regulatory genes were downregulated. *Rad52*, *ATM*, *ERCC5*, *PPIC*, *KAT2B*, and notably *FOXO4* showed a significant increase. A very similar pattern was observed in the sequencing data after a combined treatment of crizotinib and C-ions IR. The particle irradiation with 4 Gy C-ions showed no significant changes in these genes, confirming the poor radiosensitivity of this tumor entity. Venn analysis identified *FOXO4*, *CDC20*, *EXO1*, and *Rad52* in MUG-Chor1 cells and *FOXO4* and *CDC20* in U-CH2 cells that were significantly affected by all treatment regimes.

p53 and the forkhead box O (FOXO) family share similar biological functions, particularly in regulating the balance between cell death and survival, thereby restricting cell proliferation in stressed cells [31, 32]. The p53(*TP53*)-*FOXO4* axis play a crucial role in the induction of cellular senescence, which is a viable, non-proliferative state that differs from both quiescence and permanent differentiation. *FOXO4* interacts directly with p53 in the cell nucleus, thereby preventing p53 from translocating to the mitochondria and triggering apoptosis. As a result, damaged cells are not eliminated but remain in a senescent state [33]. Ionizing radiation-induced senescence in human fibroblasts leads to elevated *FOXO4* expression [34]. In this context, the significant upregulation of *FOXO4* expression in sacral chordoma cells after crizotinib, respectively, the combined treatment with C-ions irradiation appears to reflect an increase in senescence. Both the crizotinib treatment alone and the irradiation with C-ions significantly arrested chordoma cells in the G_2_/M phase. Interestingly, after combined treatment, there is a reversal of the radiation effect. Additionally, a significantly reduced expression of the proliferation markers cMyc, survivin, and *PCNA* can be observed.

During the DNA damage response, IR-induced DNA double-strand breaks activate ATM in a complex interplay, which directly phosphorylates p53 and also phosphorylates and activates the checkpoint kinases CHK1 and CHK2. This mechanism safeguards p53 from *MDM2*-mediated ubiquitination and subsequent proteasomal degradation, whereby *FOXO4* can directly interact with *MDM2* [35, 36]. Under our experimental conditions, C-ions irradiation activated p-p53 in a dose-dependent manner. However, this radiotherapy-induced activation was diminished when combined with crizotinib treatment. The increased phosphorylation of DNA-PKcs after combined treatment with crizotinib activates, as a central component of the Non-Homologous End Joining (NHEJ) repair pathway for DNA double-strand breaks (DSBs), an efficient damage repair response and thereby promotes enhanced radioresistance. In contrast, partial inhibition of the master kinase ATM, together with downstream signaling via pCHK2 and p-p53, results in a reduced repair capacity that can be interpreted as radiosensitization. A reduction in pCHK1 activity further supports this interpretation, as it induces checkpoint failure, allowing cells with unrepaired damage to enter mitosis. This is consistent with the results of FACS cell cycle analyses, which demonstrate a reversal of the radiation effect and indicate mitotic catastrophe as well as increased radiosensitivity. However, these relatively weak effects induced by the combined treatment with crizotinib are not sufficient to significantly enhance the efficiency of γH2AX phosphorylation.

Crizotinib occupies the ATP-binding site of receptor tyrosine kinases, thereby inhibiting their autophosphorylation and subsequent activation of downstream pathways, including PI3K/AKT/mTOR and RAS/RAF/MEK/ERK [37]. Both crizotinib monotherapy and its combination with C-ion irradiation strongly diminish AKT and ERK phosphorylation in sarcal chordoma cells and resulted in a significant downregulation of important ATM downstream genes, including *BRCA1*, *SMC1A*, *FANCD2*, and *cdc25c*, while the expression of *CREBBP* and *MDM2* was elevated.

The ALK/MET inhibitor crizotinib is a promising cancer therapy used primarily for the treatment of non-small cell lung cancer [37], and demonstrated cytotoxic effects on cervical cancer [38], ovarian cancer [39], and also chordoma cell [12, 13]. While combination therapy with C-ions radiation produced notable effects, it fulfilled our expectations regarding radiosensitization only to a limited degree. Baschnagel et al. reported similar findings in head and neck squamous cell carcinoma, observing minimal or no increase in radiosensitivity [40].

In summary, C-ions irradiation caused a slight dose-dependent reduction in proliferation, a clear G_2_/M cell cycle arrest, and a significant dose-dependently activation of the key regulators of the DNA repair and damage response in human sacral chordoma cells. The ALK/MET inhibitor crizotinib, as a potential therapeutic agent for chordomas, led to a reduction of proliferation markers and the modulation of key DNA repair and cell cycle regulatory genes, with *CDC20* and *FOXO4* playing a pivotal role. Protein phosphorylations of key regulators involved in DNA repair and damage prevention, as well as MAPKs activated by C-ions irradiation, were partially deactivated by combined treatment with crizotinib. Crizotinib shows potential as a therapeutic agent for treating sacral chordomas; however, its ability to enhance radiosensitivity is limited.

## Supplementary Information

Below is the link to the electronic supplementary material.

Supplementary Material 1

## Data Availability

All data supporting the findings of this study are available within the paper and its Supplementary Information.
