# Supplementary material for: Evaluation of crizotinib as radiosensitizer in sacral chordoma cells: effects of combined carbon ion particle therapy
Source: Med Oncol. 2025 Dec 24;43(2):59. doi: 10.1007/s12032-025-03172-8 (PMC12738640; doi:10.1007/s12032-025-03172-8)
Supplement: Supplementary file 1 — Supplementary Material 1 [file 12032_2025_3172_MOESM1_ESM.pdf]

Evaluation of Crizotinib as Radiosensitizer in Sacral Chordoma Cells: Effects of Combined Carbon Ion Particle Therapy

Birgit Lohberger[1*], Dietmar Glänzer[1], Vanessa Etschmaier[1], Slave Trajanoski[2], Andreas Leithner[1], Beate Rinner[3], Dietmar Georg[4,5]

[1] Department of Orthopedics and Trauma, Medical University of Graz, Graz, Austria;

[2] Core Facility Computational Bioanalytics, Medical University of Graz, Graz, Austria;

[3] Division of Biomedical Research, Medical University of Graz, Graz, Austria;

[4] Department of Radiation Oncology, Medical University of Vienna, Vienna, Austria;

[5] MedAustron-Ion Therapy Center, Wiener Neustadt, Austria

***corresponding author**: ResProf. PD Dr. Birgit Lohberger, MSc (ORCID: 0000-0002-3156-7902); Department of Orthopedics and Trauma, Medical University Graz; Auenbruggerplatz 29, A-8036 Graz; email: birgit.lohberger@medunigraz.at

Table S1. Cell cycle distribution of chordoma cell lines 48 h after IR with 0 Gy (non-irradiated control cells), 2 Gy, 4 Gy, and 8 Gy C-ions. Asterisks represent significant differences between control and irradiated cells (*$p < 0.05$; **$p < 0.01$; ***$p < 0.005$; $n = 5$; mean ± S.D.)

	C-ion IR	***G_1/G_0***	***S***	***G_2/M***
MUG-Chor1	0 Gy	66.22±1.9	22.54±4.2	11.24±4.1
U-CH2	0 Gy	68.14±7.3	17.9±5.1	13.96±8.2
MUG-Chor1	2 Gy	45.37±3.1 ***	14.58±3.2 *	40.05±0.8 ***
U-CH2	2 Gy	50.40±3.46	15.81±7.2	33.78±8.4 *
MUG-Chor1	4 Gy	29.51±5.8 ***	14.08±5.3 *	56.42±8.7 ***
U-CH2	4 Gy	34.79±8.2 **	20.03±9.8	45.18±6.3
MUG-Chor1	8 Gy	16.46±1.9 ***	17.23±8.0	66.30±8.9 ***
U-CH2	8 Gy	29.93±8.9 **	26.72±10.1 *	43.34±3.2 ***

Table S2. Cell cycle distribution of chordoma cell lines 48 h after IR with 0 Gy (ctrl 0 Gy), cells treated with 5 µM crizotinib (Crizo 0 Gy), irradiated with 4 Gy C-ions (C-ions 4 Gy), or subjected to the combined treatment (Crizo 4 Gy). Asterisks represent significant differences between control and irradiated cells (*p < 0.05; **p < 0.01; ***p < 0.005; n = 5; mean ± S.D.). Rhomboids describe the significant differences between 4 Gy C-ions and the combined treament with crizotinib (#p < 0.05; ##p < 0.01; ###p < 0.005).

	C-ion IR	***G_1/G_0***	***S***	***G_2/M***
MUG-Chor1	ctrl 0 Gy	44.18±7.7	28.92±3.8	26.89±7.4
U-CH2	ctrl 0 Gy	46.14±8.3	17.47±7.8	26.11±5.9
MUG-Chor1	Crizo 0 Gy	54.11±4.2 *	17.85±4.7 **	28.03±4.0
U-CH2	Crizo 0 Gy	47.38±5.2	27.32±4.2	25.26±8.5
MUG-Chor1	C-ions 4 Gy	17.23±1.8 **	30.22±5.0	52.54±5.9 ***
U-CH2	C-ions 4 Gy	25.84±2.7 *	16.35±6.6	57.81±4.3 ***
MUG-Chor1	Crizo 4 Gy	55.27±3.9 * ###	20.46±5.6 * #	24.26±3.1 ###
U-CH2	Crizo 4 Gy	46.47±4.9 ##	28.89±5.7 #	24.66±6.5 ###

uncropped files Figure 1c

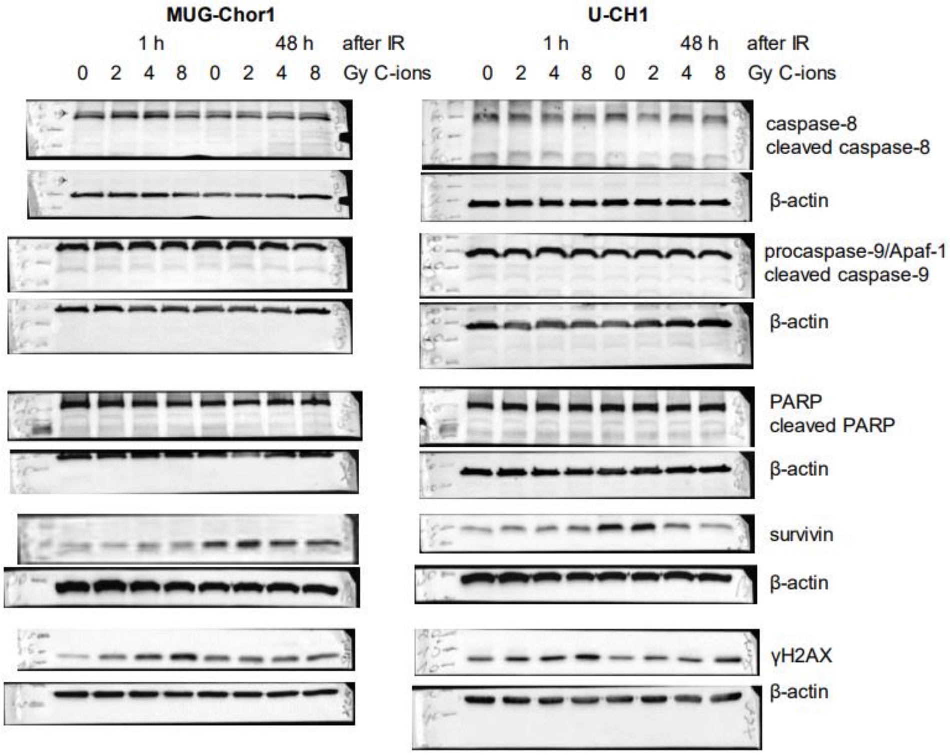

uncropped files Figure 1d

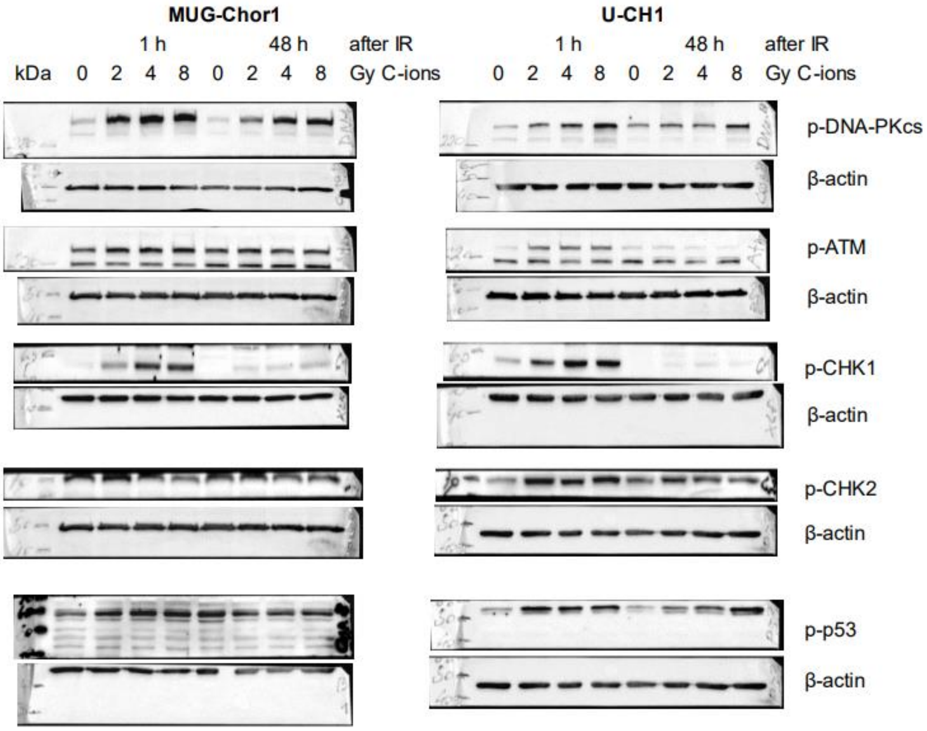

uncropped files Figure 5a

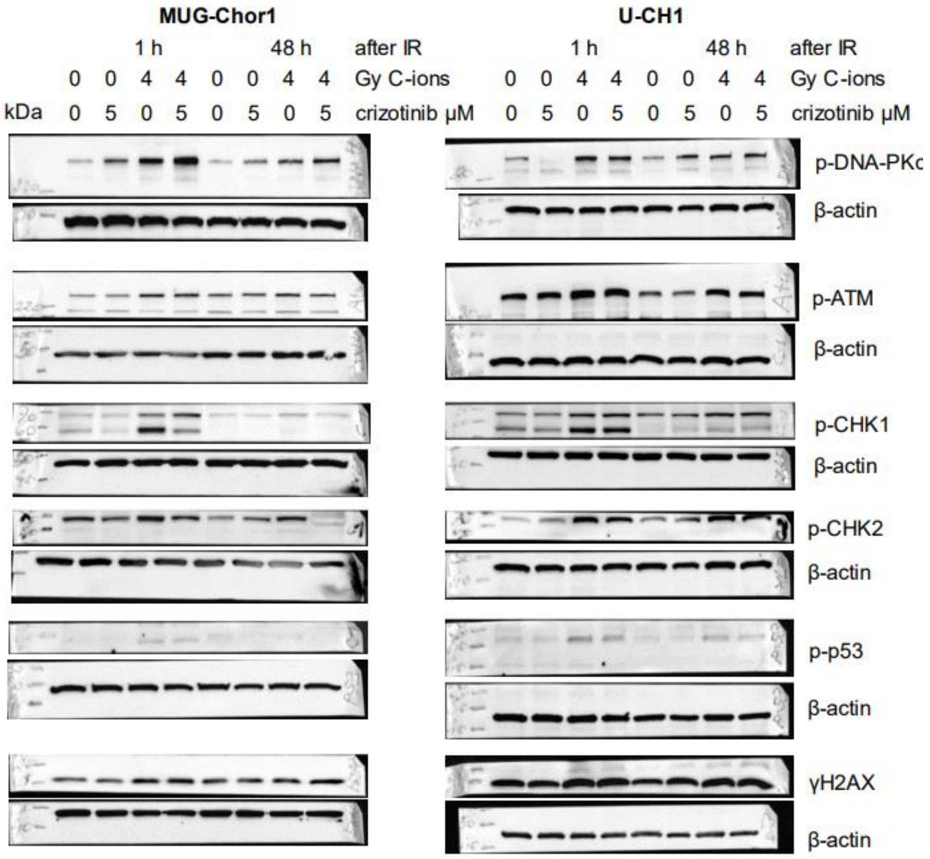

uncropped files Figure 5b

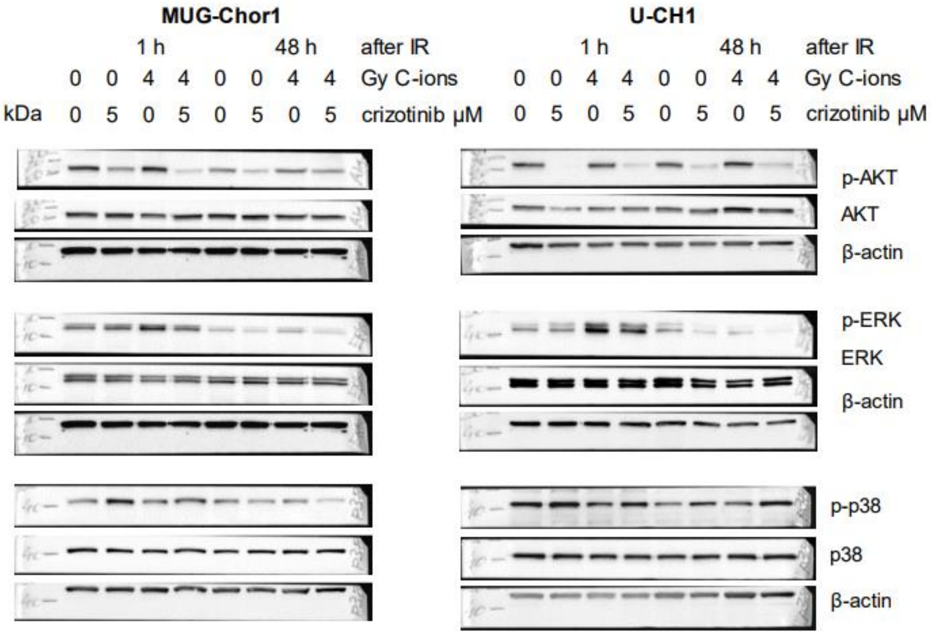

Fig. S1 Full-length blots from all western blots are presented.
